# Identification of the prognostic and immunological roles of aquaporin 4: A potential target for survival and immunotherapy in glioma patients

**DOI:** 10.3389/fncel.2022.1061428

**Published:** 2022-11-29

**Authors:** Yu-Long Lan, Tian Nie, Shuang Zou

**Affiliations:** ^1^Department of Neurosurgery, The Second Affiliated Hospital Zhejiang University School of Medicine, Hangzhou, Zhejiang, China; ^2^Department of Neurology, The Second Affiliated Hospital Zhejiang University School of Medicine, Hangzhou, Zhejiang, China; ^3^Key Laboratory of Precise Treatment and Clinical Translational Research of Neurological Diseases, Hangzhou, Zhejiang, China; ^4^Clinical Research Center for Neurological Diseases of Zhejiang Province, Hangzhou, China; ^5^Department of Neurology, Affiliated Hangzhou First People’s Hospital, Zhejiang University School of Medicine, Hangzhou, Zhejiang, China; ^6^Department of Pharmacy, Zhejiang Chinese Medical University, Hangzhou, Zhejiang, China

**Keywords:** aquaporin 4, glioma, immune, target, treatment

## Abstract

Recent studies have revealed the critical role of *AQP4* in the occurrence and development of gliomas. However, the role of *AQP4* in immune regulation has not yet been reported. Many recent reports have identified the lymphatic system’s occurrence within the central nervous system (CNS) and the vital role of immune regulation in treating brain tumors. Therefore, the present study aimed to explore the role of *AQP4* in the immune regulation of glioma. We used bioinformatics analysis to investigate the immunoregulatory function of *AQP4*, including its correlation with immunity, anti-tumor immune processes, immunotherapy, immune infiltration, tumor mutational burden (TMB), stemness, mutation, and pan-cancer. The results revealed that *AQP4* was significantly associated with the expression of multiple immune checkpoints, immune cells, as well as multiple immune cell effector genes, and antigen presentation and processing abilities. Although no significant correlation was found between the *AQP4* gene and IDH mutation and *MGMT*, *AQP4* demonstrated substantial expression differences in different immunophenotypes and molecular types. Using the TTD database, we discovered that EGFR, ABAT, and PDGFRA are strongly associated with *AQP4* expression in the glioblastoma (GBM) classification, and these factors could be the potential *AQP4*-related immunotherapy targets. Afterward, we screened the differential genes in the high and low *AQP4* gene expression group, the high and low immune score group, and the high and low matrix score group and took the intersection as the candidate factor. Finally, univariate Cox analysis was used to find eight prognostic variables with significant differences across the candidate genes. After lasso dimensionality reduction, three genes built the model (RARRES1, SOCS3, and TTYH1). The scoring model generated by the three genes was eventually obtained after the multi-factor screening of the three genes. Finally, combined with clinical information and cox regression analysis, it was further confirmed that the model score could be used as an independent prognostic factor.

## Introduction

Glioma is the most common primary central nervous system (CNS) tumor, accounting for about 60–70% of primary brain tumors, and the overall survival of patients after diagnosis is about 15–18 months (Smith et al., 2022). Glioblastoma (GBM) is the most malignant type. GBM is a major problem in the field of neurosurgery at present. The main treatment methods are surgery, combined with radiotherapy and chemotherapy. The treatment effect is poor due to its rapid development, high invasiveness, and resistance to chemotherapeutic medications, and novel treatments are urgently needed to improve patient prognosis. Simultaneously, if novel treatment targets can be discovered, they will undoubtedly benefit most GBM patients.

Currently, 13 aquaporins (*AQP0–AQP12*) have been identified in mammals, of which *AQP4* is a class of aquaporins expressed explicitly in the CNS. A significant number of neurological disorders are linked to changes in *AQP4* expression or location. *AQP4* plays a role in brain inflammation, glial lymphoid clearance, synaptic plasticity, memory development, extracellular space (ECS) volume modulation, and potassium homeostasis (Papadopoulos and Verkman, 2012; Nagelhus and Ottersen, 2013; Hubbard et al., 2018). The research on *AQP4* and various diseases is mainly based on the pathological analysis of dead brain tissue, *in vitro* experimental research, and the use of *AQP4*-deficient rodent models (Mader and Brimberg, 2019). Only a few studies use extensive data analysis, and bioinformatics research on *AQP4* will assist in a more systematic and thorough investigation of *AQP4*’s role in numerous disorders.

The role of *AQP4* in the occurrence and development of glioma is still unclear. Our earlier study systematically summarized the critical role of *AQP4* in the malignant progression of glioma and its significance in the study of anti-tumor drug resistance (Lan et al., 2017). In glioma, the expression of the AQP4 protein is elevated, and inhibiting *AQP4* can significantly reduce glioma malignant proliferation (Kahlert et al., 2013). The most recent research indicates that temozolomide can suppress the growth of malignant glioma by inhibiting the expression of *AQP4*, implying that the *AQP4* pathway’s activity substantially impacts the chemotherapeutic effectiveness of GBM (Chen et al., 2017). Our two newly published studies further confirmed the great potential of *AQP4* in treating GBM (Lan et al., 2020; Zou et al., 2021). We found for the first time that inhibition of *AQP4* can significantly improve the sensitivity of GBM drug therapy (Lan et al., 2020). More intriguingly, current research has indicated that AQP4 protein aggregation state could be a determinant for glioma cell fate (Amiry-Moghaddam, 2019; Simone et al., 2019), strengthening the potential role of *AQP4* as a target in the treatment of GBM. It’s known that AQP4 forms heterotetramers in the plasma membrane made of the M23-AQP4 and M1-AQP4 isoforms (Smith and Verkman, 2015). The isoform ratio controls AQP4 protein aggregation into supramolecular structures called orthogonal arrays of particles (AQP4-OAP). The role of AQP4 protein aggregation into OAP in malignant gliomas is still unclear. The authors (Simone et al., 2019) found that AQP4 protein aggregation/disaggregation into OAP influences the biology of glioma cells, demonstrating that AQP4 protein disaggregation may potentiate invasiveness potential, whereas AQP4 protein aggregation may activate the apoptotic path. Besides, Valente et al. (2022) found that AQP4ex, the new read through isoform of AQP4, could be associated with the degree of vasogenic brain edema and GBM cell progression. They found that the reduction in AQP4ex, leading to reduction and delocalization of AQP4, could be likely to undermine the integrity of the BBB, indicating that AQP4ex could be considered as a potential new early biomarker of GBM progression and a target for AQP4 modulation (Frigeri et al., 2007). Various studies have confirmed that *AQP4* plays a vital role in GBM’s malignant progression and drug resistance (Valente et al., 2022), but its molecular mechanism still needs more in-depth and comprehensive research (Peng et al., 2020; Behnam et al., 2022).

Tumor immunotherapy is a prominent topic in anti-tumor research, and the significance of *AQP4* in immune modulation has yet to be discovered. Our previous research has indicated that *AQP4* exerted carcinogenic effects *via* various pathways in glioma (Lan et al., 2017). Furthermore, regulatory T-cell development has been found to be dependent on *AQP4* expression (Chi et al., 2011). Mice lacking AQP4 receptors had suppressed levels of CD4^+^/CD25^+^ regulatory T-cells. This leads to an abnormally overactive microglial inflammatory response (Chi et al., 2011). In recent years, many reports have identified the occurrence of the lymphatic system within the CNS and its essential role in immune regulation in brain tumor therapy (Lan et al., 2022; Li et al., 2022). As a result, the involvement of *AQP4* in the immune regulation of glioma and the immunotherapy process was investigated further in this work. This study aimed to examine the role of *AQP4* in the CNS immune system and find out how important it is in the glioma immunotherapy process. More importantly, we anticipate developing an *AQP4*-related prognostic model, which would serve as a critical theoretical research foundation for improving the effect of glioma immunotherapy.

## Materials and methods

### Data retrieval and preprocessing

Expression profile data, phenotype data, and mutation data of GBM were downloaded from the Xena^1^ database. The GBM data packet named “01A,” which including 114 samples (Table 1), was selected. The data itself has been log_2_(data + 1). The information uses FPKM (Fragments Per Kilobase of transcript per Million mapped reads) without the normalization between arrays of the limma package. Twenty immune checkpoints and 122 immunomodulator-related genes were obtained from the document “Siglec15 shapes a non-inflamed tumor microenvironment and predict the molecular subtype in bladder cancer.” GBM data were obtained from CGGA (Chinese Glioma Genome Atlas) database. Data samples from two different data packets contain 693 cancer data and 325 cancer data, respectively. The number of GBM and rGBM in data packet of 693 data samples is 209, and the number of GBM and rGBM in data packet of 325 samples is 109. The raw data is RSEM, which has been converted to log2(data + 1).

**TABLE 1:** TCGA patient information included in the study.

Variable	TCGA cohort (*n* = 144)	
	*n*	%
**Age**		
<60	67	46.53%
≥60	76	52.78%
**Gender**		
(1) Male	95	65.97%
(2) Female	48	33.33%
**Immune subtype**		
(1) Immune C1	3	2.08%
(2) Immune C4	139	96.53%
(3) Immune C5	1	0.69%
**IDH status**		
(1) WT	131	90.97%
(2) Mutant	10	6.94%
**MGMT promoter status**		
(1) Unmethylated	68	47.22%
(2) Methylated	46	31.94%
**Original subtype**		
(1) Mesenchymal	46	31.94%
(2) Neural	25	17.36%
(3) Classical	34	23.61%
(4) Proneural	28	19.44%
(5) G-CIMP	8	5.56%

### Correlation analysis between *AQP4* gene and immune checkpoints

The Hmisc package of R was used to calculate the correlation analysis of 20 immunological checkpoints in the data of the gene AQP4 and TCGA. Spearman’s technique was utilized for correlation analysis.

### Correlation between *AQP4* gene and immune infiltration

The R’s CIBERSORT was applied to assess the proportion of infiltrating 22 immune cells in cancer samples. The correlation between the gene *AQP4* and the 22 immune cells implanted ratio was calculated. The same analysis was performed in low grade glioma (LGG), GBM, LGG + GBM, lung squamous cell carcinoma, lung adenocarcinoma, and breast cancer.

### Differential analysis of immunomodulator-related genes between high and low *AQP4* gene expression groups

The limma package in R was used to do the difference analysis. The median expression of the *AQP4* gene served as the grouping criterion; individuals with expression levels higher than the median were classified into high expression groups, while those with expression levels lower than the median were divided into low expression groups. The screening criteria for differential genes were *P*-value < 0.05, |log2FC| > 0.5853. The differential genes were intersected with 122 immunomodulator-related genes to obtain differential immunomodulatory genes, plotted by R’s heatmap package.

### The anti-cancer immune process activity is different in the high and low expression of the *AQP4* gene

Immune circulation data for GBM were obtained from the TIP (Trip Medical Database) database and plotted using the ggplot2 package for R.

### *AQP4* gene-related functional enrichment analysis

The differential genes of *AQP4* high and low groups were analyzed for functional enrichment by the cluster profile package of R.

### Heatmap of the relationship between *AQP4* gene high and low groups and tumor-associated immune cell effector genes

Heatmaps of tumor-associated immune cell effector genes were created using the R package pheatmap and data from the literature “Siglec15 shapes a non-inflamed tumor microenvironment and predict the molecular subtype in bladder cancer.”

### Differential expression of *AQP4* gene in clinical features

The patients were grouped according to clinical characteristics, including age, sex, immunophenotype, molecular type, IDH mutation, and MGMT, and the expression of the *AQP4* gene in different groups was compared.

### Correlation of *AQP4* gene expression with tumor mutational burden, immune score, stromal score, and tumor purity

Immune scores, stromal scores, and tumor purity were calculated through the estimate package for R. The R package maftools were used to calculate TMB. The R package Hmisc was then used to calculate the connection between the expression value of gene *AQP4* and TMB, immunological score, stromal score, and tumor purity.

### *AQP4* gene high and low expression groups, enrichment scores of immunotherapy prediction-related pathways, and differences in *AQP4* gene expression among different immune response groups in the immune dataset

The R’s GSVA package was used to perform the pathway enrichment scores of *AQP4* gene high and low groups, and the *AQP4* gene expression in different immune groups in IMvigor210 (bladder cancer) and GSE91061 (melanoma) were analyzed. GSE126045 (NSCLC) was excluded from data analysis due to a lack of data.

### Correlation analysis of *AQP4* gene mutation, copy number variation, methylation, and expression

The number of *AQP4* mutations is low. Copy number variation and methylation data were obtained from the Xena database.^2^

### Mutations in *AQP4* gene expression high and low groupings

The GenVisR package for R displayed high and low-grouped mutations in *AQP4* gene expression.

### Database screening for differential expression display of cancer-type therapeutic targets

Screening of cancer-type therapeutic targets was performed using data from the TTD (Therapeutic Target Database) database.^3^

### Screening the intersection of differential genes of *AQP4* gene expression group, immune score group, and matrix score group as candidate factors

As possible factors, the intersection of differential genes in high and low *AQP4* gene groups, differential genes in high and low immunological score groups, and differential genes in high and low matrix score groups was used. The R package used is limma, and the filtering conditions are *P-*value < 0.05, |log2FC| > 0.585.

### Screening of prognostic factors by univariate cox regression based on differential genes

The intersection of the above differential genes was subjected to single factor cox analysis by R survival. For subsequent analysis, genes with *P*-values less than 0.05 were screened as prognostic factors.

### Lasso dimensionality reduction removes redundant factors and builds a related prognostic risk scoring model

Lasso analysis was performed by R’s glmnet package, followed by multivariate cox regression to obtain model scores.

### Evaluation of the predictive power of prognostic models

The survival package in R was used to perform KM (Kaplan-Meier) survival analysis on the models. The survival ROC package in R was used to examine the AUC value of the model score and the different analyses of the genes in the high and low model score groups.

### Validation of prognostic models with external datasets

The above analysis was carried out using ICGC (The International Cancer Genome Consortium) data.

### Univariate and multivariate cox regression analyses

Combined with clinical information, univariate and multivariate cox regression analysis verified that risk scores were independent prognostic factors (binary classification method, at least stage, age, and gender were included). Univariate and multivariate separate predictive analyses were performed on risk scores.

### Combined with clinical characteristics, a nomogram was developed to assist in clinical practice

The R’s rms package, foreign, and survival packages were used to construct a nomogram for clinical features and risk scores.

### Human tissue microarray and immunohistochemistry

The human tissue microarrays were purchased from Outdo Biotech Company (Shanghai, China). A total of 77 glioma tissue samples and three normal samples were included. Slides were deparaffinized and rehydrated and were then immersed in target retrieval solution (pH 6) and boiled at medium heat three times for 10 min each in a microwave. After the slides were blocked with 3% BSA, the sections were incubated with primary antibody against AQP4 (Santa Cruz Biotechnology, Dallas, TX, USA) followed by HRP-labeled anti-rabbit IgG secondary antibody. The specimens were counterstained with hematoxylin. The negative control was obtained by replacing the primary antibody with a regular rabbit IgG. Target-positive cells were counted in 3-4 different fields and imaged using an Olympus microscope, and the immunoreactions were evaluated independently by two pathologists blinded to the clinicopathologic information in order to ensure unbiased assessment of tissue morphology. The antibody staining intensity was classified as follows: no staining, 0; weak staining, 1; moderate staining, 2; and strong staining, 3. A five-point scale was used to classify the percentage of cells stained: 0 (no positive cells), 1 (<25% positive cells), 2 (25–50% positive cells), 3 (50–75% positive cells), and 4 (>75% positive cells). The score for each tissue was calculated by multiplying the intensity index by the percentage index, yielding a score between 0 and 12. The median AQP4 score was employed to determine the cutoff value. Tumors with AQP4 scores less than or equal to the median were designated as having “low expression,” whereas those with scores greater than the median were designated as having “high expression.”

### Statistical analysis

The Spearman test was utilized in every correlation analysis. The ns denotes that there is no significant difference, * means *p*-value < 0.05, ^**^ means *p*-value < 0.01, ^***^ means *p*-value < 0.001, ^****^ means *p*-value < 0.0001.

## Results

### AQP4 protein expression may predict prognosis in glioma patients

**FIGURE 1:**
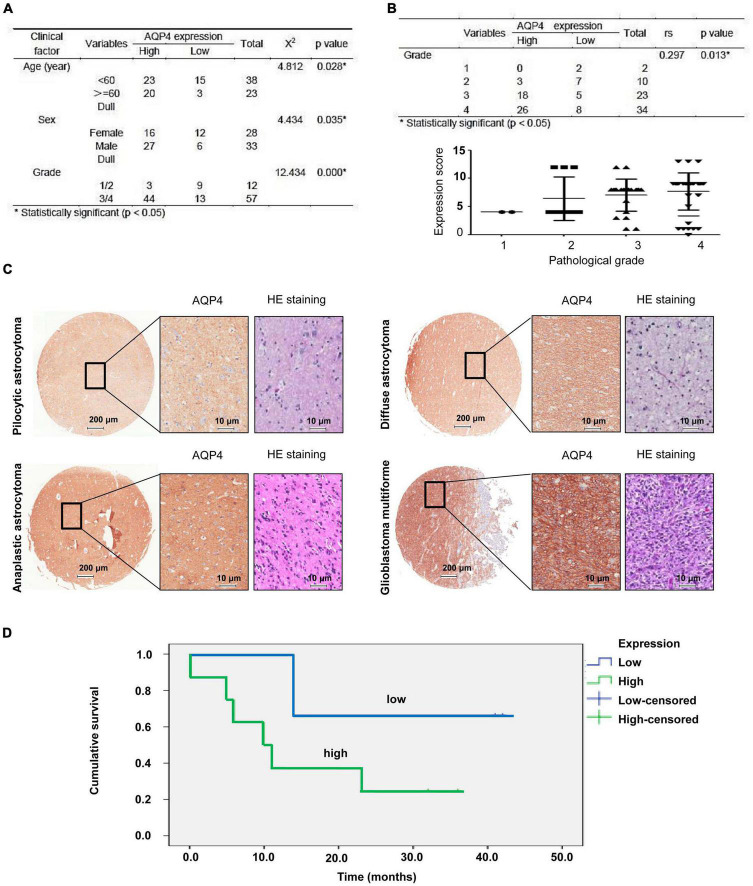
Clinical data analysis of AQP4 expression in human tissue microarrays from glioma patients. **(A)** Correlation analyses of AQP4 protein expression in relation to clinicopathologic variables of 77 glioma patients by a X ^2^ test. **(B)** Quantitative analysis of the relative AQP4 expression level in all groups was performed. Furthermore, the correlation between AQP4 expression and different glioma grades was also assessed by Spearman’s rank correlation analysis. **(C)** Representative images of immunohistochemical staining to assess AQP4 protein levels in the glioma tissue microarray. **(D)** Kaplan-Meier analysis of OS for glioma cancer patients with different expression levels of AQP4 by a log-rank test. “Low” indicates a low level of AQP4 expression; “high” indicates a high level of AQP4 expression.

The expression of AQP4 protein in tumor samples from 77 glioma patients was evaluated by immunohistochemical (IHC) staining of a tissue microarray. The expression of AQP4 protein in each IHC sample was classified as either high (score > 6) or low (score ≤ 6), using the median of the IHC scores as the cutoff value. The correlations between AQP4 protein expression and the clinicopathologic variables of 77 glioma patients are shown in Figure 1A. The results indicated that low AQP4 expression was significantly correlated with lower clinical grade glioma (grade 1/2), while high AQP4 expression predicted higher clinical grade glioma (grade 3/4) in patients (Figure 1A) (X ^2^ = 12.434, *p* < 0.001). In addition, younger age was significantly correlated with low expression of AQP4 (X ^2^ = 4.812, *p* = 0.028), and female patients tended to have lower expression of AQP4 than male patients (X ^2^ = 4.434, *p* = 0.035). More importantly, we also found that AQP4 was highly expressed in glioma tissues and that the expression level of AQP4 was negatively correlated with the glioma grade (Figures 1B,C) (Spearman’s rank correlation rs = 0.297, *p* = 0.013). Kaplan-Meier OS analyses revealed that the low AQP4 expression group (IHC staining indicating strong and moderate expression) had a better prognosis than the high AQP4 group (IHC staining indicating weak and negative expression) (*p* < 0.01) (Figure 1D). Overall, these data demonstrated the prognostic significance of AQP4 in glioma.

### Correlation of *AQP4* gene with mainstream immune checkpoints and immune infiltrating cells

**FIGURE 2:**
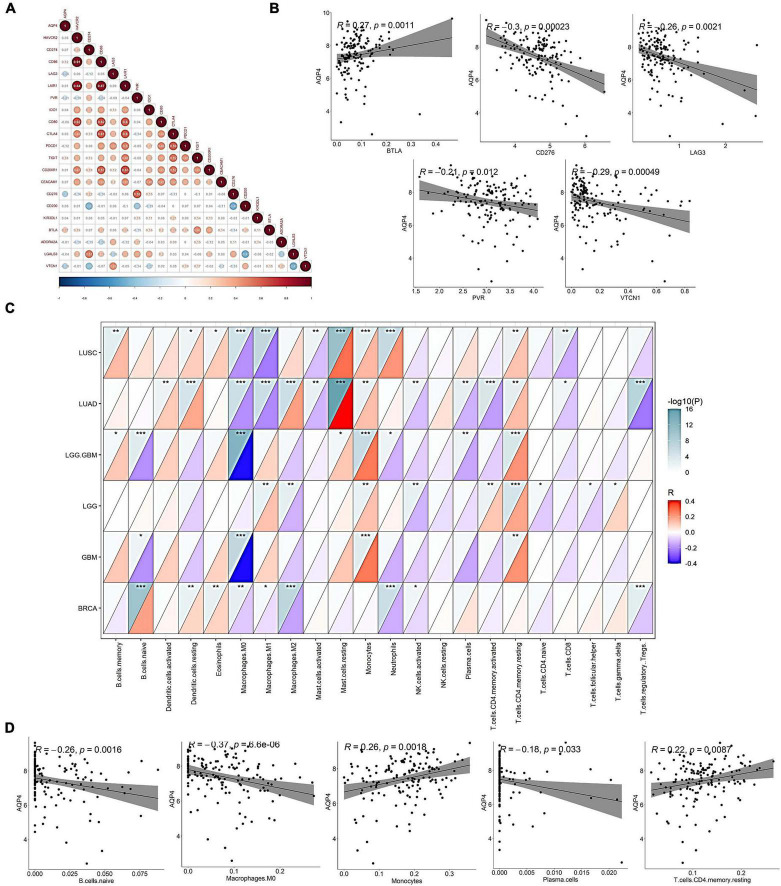
Correlation of *AQP4* gene with mainstream immune checkpoints and immune infiltrating cells. **(A)** Association analysis of the gene *AQP4* with 20 immune checkpoints. **(B)** Correlation analysis of *AQP4* gene with mainstream immune checkpoints CD276, LAG3, PVR, BTLA, and VTCN1. **(C)** Correlation analysis of *AQP4* gene with immune infiltrating cells in pan-cancer. **(D)** Correlation analysis of *AQP4* gene with immune infiltrating cells, B cells. Naive macrophages M0, plasma cells, T-cells, CD4, memory, resting, and monocytes. *p < 0.05, **p < 0.01, ***p < 0.001.

In the correlation analysis between the gene *AQP4* and 20 immune checkpoints, only five immune checkpoints were significantly correlated. CD276, LAG3, VTCN1, and PVR were negatively correlated with the gene *AQP4*, and BTLA was negatively correlated with the gene *AQP4* (Figures 2A,B and Supplementary Tables 1–3), and the correlations were all statistically significant. We also looked into the relationship between *AQP4* expression and the number of immune invading cells (Figures 2C,D). The *AQP4* gene was strongly connected with five immune cells, including B cells, out of 22 immune invading cells. Macrophages are immature. M0, plasma cells, T-cells, CD4 memory resting, and monocytes are some terms used to describe M0, plasma cells, T-cells, and monocytes (Supplementary Tables 4–6). The tumor types we included in the study mainly included LGG, GBM, LGG + GBM, lung squamous cell carcinoma, lung adenocarcinoma, and breast cancer. In Macrophages M0, all cancers except LGG were significantly associated with the gene *AQP4*. Only breast cancer had no significant correlation with the gene *AQP4* in monocytes and T-cells, memory resting. In contrast, LGG, GBM, LGG + GBM, lung squamous cell carcinoma, and lung adenocarcinoma all had significant correlations (Supplementary Tables 7, 8).

### The effect of *AQP4* expression level on the anti-tumor immune process

**FIGURE 3:**
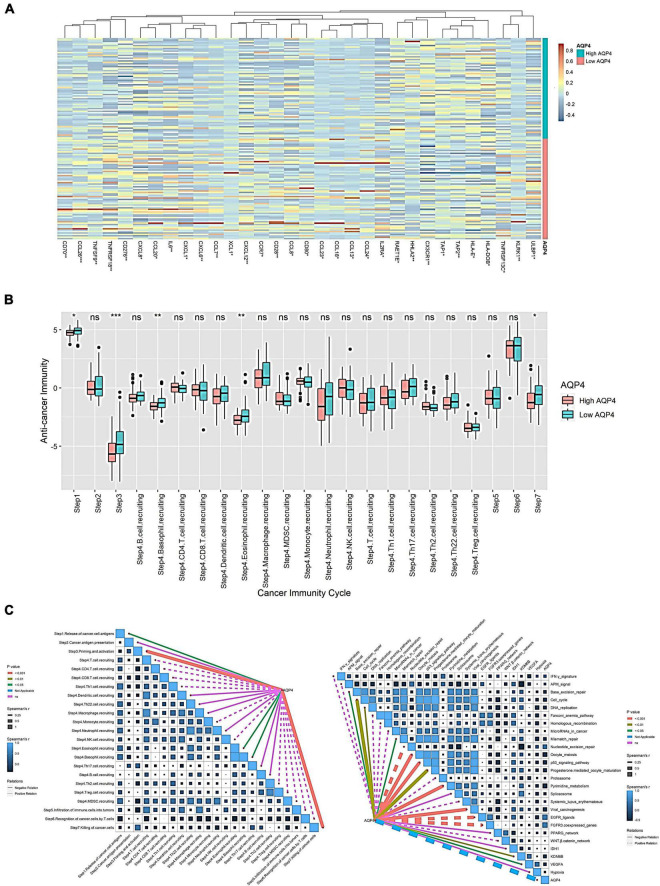
Effect of *AQP4* expression level on immune cell anti-tumor immune process. **(A)** Differential gene heatmap of immunomodulator-related genes in high and low *AQP4* expression groups. **(B)** The anti-cancer immune process activity was grouped differently in the high and low expression of the *AQP4* gene. **(C)** Correlation analysis of *AQP4* gene with immunotherapy-related predictive pathway enrichment score and anti-cancer immune process activity. *p < 0.05, **p < 0.01, ***p < 0.001.

Initially, we looked at how *AQP4* gene expression affects immune cells’ ability to present and process antigens. A total of 122 genes were collected from the literature, 32 of which were substantially different in the *AQP4* high and low expression groups, with the majority of the differential genes being low in the *AQP4* high expression group. This indicated that antigen presentation and processing were attenuated in the high *AQP4* group (Figure 3A and Supplementary Table 9). We further analyzed the anti-cancer immune process activity differences between high and low *AQP4* gene expression groups (Figure 3B). In the high and low *AQP4* expression groups, only the following five processes were significantly different between the high and low groups, including the release of cancer cell antigens, tumor antigen presentation, initiation and activation, eosinophil recruiting, and cancer cell clearance. The other 18 immunological mechanisms, on the other hand, did not differ significantly between the high and low groups (Figure 3B and Supplementary Table 10). Following that, we examined the relationships between the *AQP4* gene, immunotherapy-related prediction pathway enrichment scores, and anti-cancer immune process activity (Figure 3C).

We obtained 26 immunotherapy-related predictive pathways from the literature. The three groups with the strongest correlation in the anti-cancer immune process activity correlation analysis are Step 3 Priming and activation, Step 7 killing cancer cells, and Step 4 eosinophil recruiting, the correlations are −0.373, −0.288, and −0.186. The enrichment score was obtained by GSVA among the immunotherapy-related prediction pathways. Finally, we found that 14 pathways were significantly correlated with *AQP4*. The three groups with the strongest correlation were FGFR3-coexpressed genes, EGFR ligands, and pyrimidine metabolism, with correlations of 0.432, 0.422, and −0.366, respectively (Figure 3C and Supplementary Table 11).

### Analysis of differential gene function, pathway enrichment, and tumor-related immune cell effector genes downstream of *AQP4* gene in glioma

**FIGURE 4:**
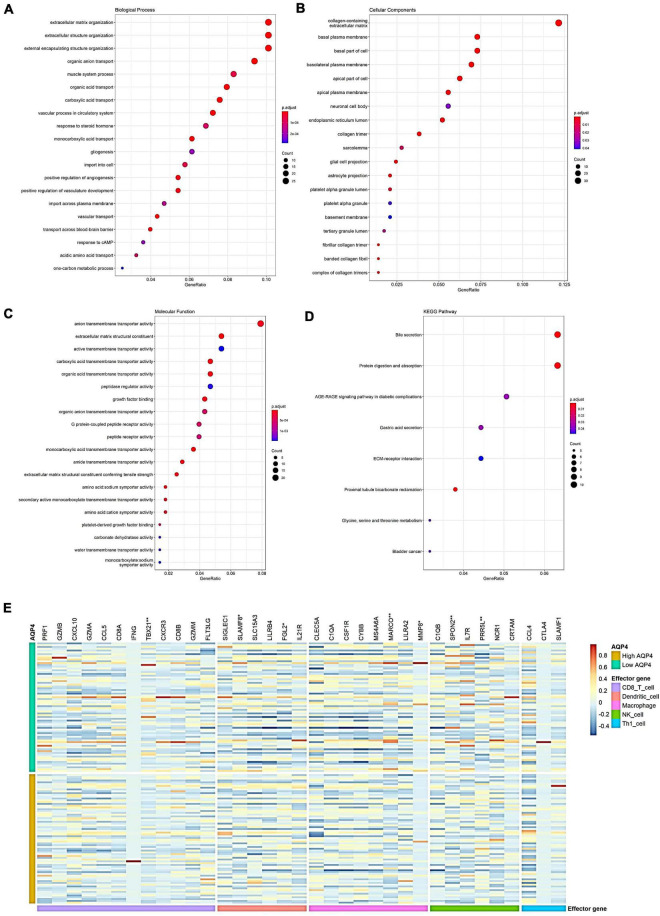
Analysis of differential gene functions, pathway enrichment, and tumor-related immune cell effector genes downstream of the *AQP4* gene in glioma. **(A–D)** Differential gene function and pathway enrichment analysis of *AQP4* gene between high and low-risk groups. **(E)** Heat map of the relationship between high and low *AQP4* gene groups and tumor-associated immune cell effector genes. *p < 0.05, **p < 0.01.

Among the biological process (BP) pathways, extracellular matrix organization, extracellular structure organization, external encapsulating structure organization, organic anion transport, and vascular process in the circulatory system have the most significant differences. The cellular component (CC) pathway showed substantial differences in the collagen-containing extracellular matrix, basolateral plasma membrane, basal plasma membrane, the basal section of the cell, and collagen trimer. In the MF pathway, the extracellular matrix structural constituents, monocarboxylic acid transmembrane transporter activity, carboxylic acid transmembrane transporter activity, organic acid transmembrane transporter activity, and growth factor binding had the most significant differences. Among the KEGG pathways, Proximal tubule bicarbonate reclamation, Bile secretion, Protein digestion and absorption, Gastric acid secretion, and AGE-RAGE signaling pathway in diabetic complications showed the most significant differences (Figures 4A–D and Supplementary Tables 12–15). The relationship between *AQP4* gene levels and tumor-associated immune cell effector genes was also investigated (Figure 4E). Seven genes were significantly altered (*p* < 0.05) among the 35 immune cell effector genes. *MARCO*, *SPON2*, *TBX21*, *MMP8*, and *SLAMF8* were among the genes with low expression in the *AQP4* high expression group, while *PRR5L* and *FGL2* were substantially expressed in the *AQP4* high expression group (Figure 4E and Supplementary Table 16).

### Effects of *AQP4* expression levels on tumor clinical features and immunotherapy targets

**FIGURE 5:**
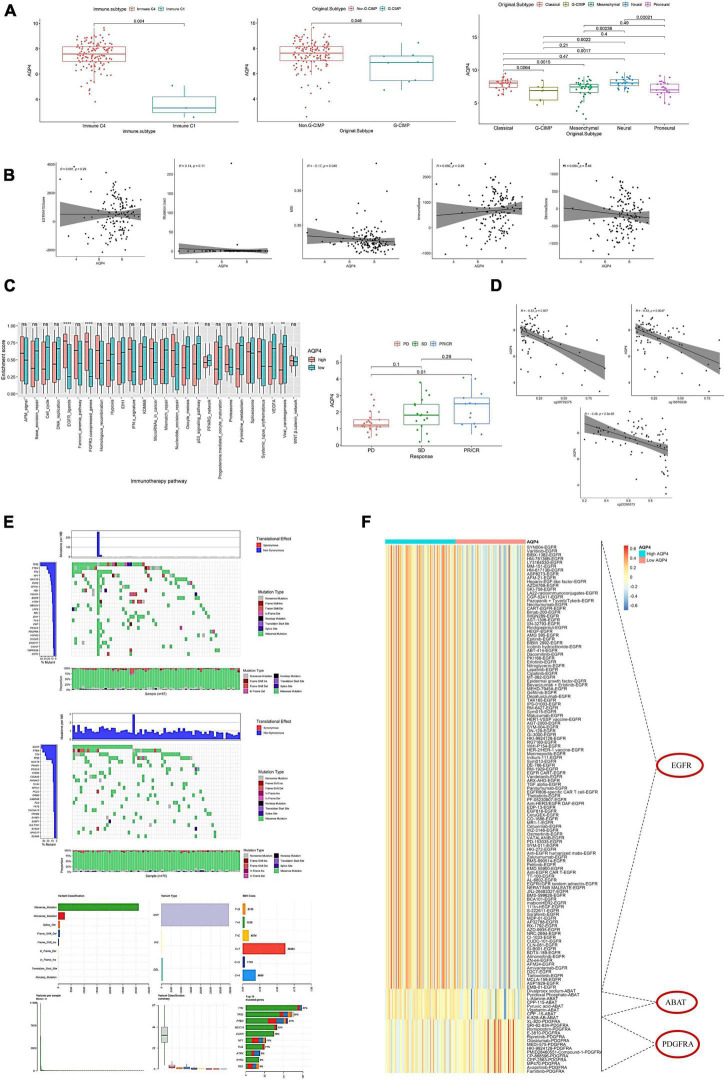
Effects of *AQP4* expression levels on clinical tumor characteristics and immunotherapy targets. **(A)** Differential expression of *AQP4* gene in clinical features. **(B)** Correlation of *AQP4* gene expression with tumor mutational burden (TMB), immune score, MSI, stromal score, and tumor purity. **(C)** Differential analysis of pathway enrichment scores for immunotherapy prediction. The left figure shows the difference in the enrichment scores of immunotherapy prediction pathways between the high and low expression groups of the *AQP4* gene, and the right figure shows the difference analysis of different immune groups in the GSE91061 dataset. **(D)** Methylation and expression correlation analysis. **(E)** *AQP4* gene expression high and low grouped mutation display. The upper figure is the *AQP4* low expression group, the middle figure is the *AQP4* high expression group, and the lower figure is the summary of the maf file. **(F)** Screening for differential expression of cancer-type therapeutic targets. *p < 0.05, **p < 0.01, ****p < 0.0001.

The *AQP4* gene did not differ significantly in age, sex, IDH mutation, and MGMT. There were significant differences between C1 and C4 in immunophenotyping. There were significant differences in molecular typing between Classical and G-CIMP, Classical and Mesenchymal, Classical and Proneural, G-CIMP and Neural, Mesenchymal and Neural, and Neural and Proneural (Figure 5A). *AQP4* gene expression was found to be substantially linked with microsatellite instability (MSI) but not with tumor mutational burden (TBI), immunological score, stromal score, or tumor purity (Figure 5B and Supplementary Table 17). In addition, we further analyzed the effect of *AQP4* gene expression level on the enrichment score of immunotherapy prediction-related pathways, as well as differences in *AQP4* gene expression among different immune response groups in the immune dataset (Figure 5C). According to the findings, only eight groups of immunotherapy predicted pathway enrichment scores were significantly different between the high and low expression groups of the *AQP4* gene. Other immunological groups exhibited significant differences in progressive disease (PD) and partial response (PR)/complete response (CR) when GSE91061 was analyzed using the *AQP4* gene (Figure 5C).

The relationship between methylation and expression of the *AQP4* gene was also investigated. *AQP4* has three methylation sites, three of which have a strong relationship with the gene’s expression (Figure 5D). We also analyzed the mutation status in the high and low *AQP4* gene expression groups and displayed the top 20 genes with mutation rates in the *AQP4* low and high expression groups (Figure 5E). In addition, we evaluated AQP4-related therapeutic targets in GBM. To extract data for analysis, we used the TDD database. The findings revealed that three genes, EGFR, ABAT, and PDGFRA, significantly differed between high and low *AQP4* expression groups in GBM (Figure 5F).

### Construction of *AQP4* gene-related clinical prognosis model

**FIGURE 6:**
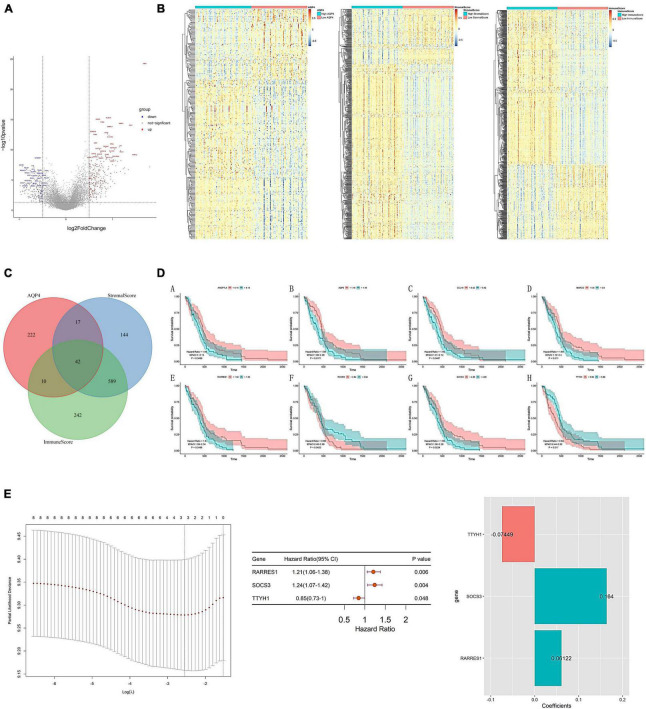
Construction of *AQP4* gene-related clinical prognosis model. **(A)** Volcano plot of differential genes between *AQP4* high and low expression groups. **(B)** The differential gene heat map of the high and low *AQP4* gene expression group, the high and low immune score group, and the high and low matrix score group. **(C)** The intersection of differential genes in high and low *AQP4* gene expression group, high and low immune score group, and high and low matrix score group. **(D)** Survival analysis of eight (A–H) prognostic factors. **(E)** Construction of a predictive risk scoring model.

As a preliminary step, we searched for possible factors in the intersection of differential genes in the *AQP4* gene high and low expression groups, the immunological score high and low group, and the matrix score group. The *AQP4* gene high and low expression group had 291 differential genes, the immunological score high and low group had 883 divergent genes, the matrix score group had 792 differential genes, and the intersection of the three groups had 42 difference genes (Figures 6A–C and Supplementary Tables 18–20). Subsequently, we performed a univariate cox analysis on the 42 differential genes and obtained eight prognostic factors with significant differences (Figure 6D and Supplementary Table 21). In addition, duplicate elements were eliminated using LASSO dimensionality reduction to develop a useful predictive risk scoring model. After lasso dimension reduction, we obtained a model consisting of three genes from the eight prognostic factors. Then perform multi-factor screening on these three genes to get the final scoring model constructed by the three genes (Figure 6E and Supplementary Table 22), that is, Risk score = RARRES1*(0.06121847) + SOCS3*(0.16404808) + TTYH1*(−0.07449025).

### Predictive efficacy evaluation of clinical prognostic models and validation on external datasets

**FIGURE 7:**
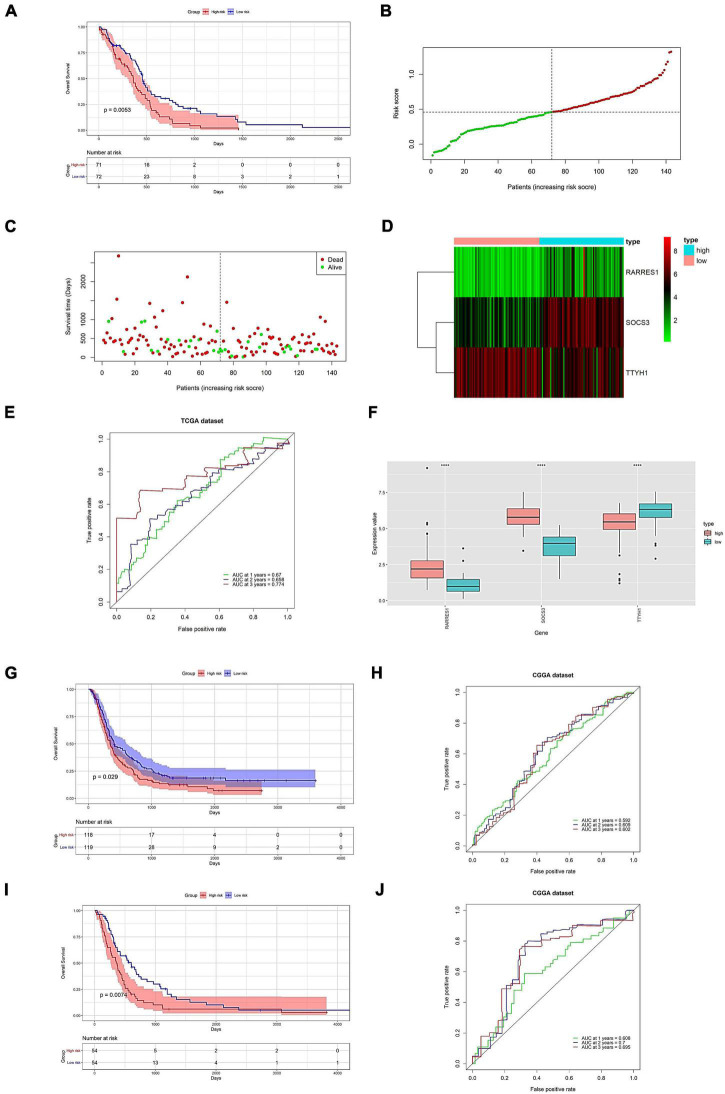
Prognostic model prediction efficacy assessment and validation on external datasets. Evaluation of predictive power **(A–F)**. **(A)** Survival analysis of high and low expression groups. **(B)** Risk score distribution. **(C)** Distribution of patient survival. **(D)** The expression of the hub gene. **(E)** Predicted ROC curve for 1–3 years. **(F)** Model genes are shown differentially in high-risk and low-risk. Validation of prognostic models on external datasets **(G–J)**. **(G,I)** Survival analysis of high and low expression groups. **(H,J)** Predicted 1-, 2-, and 3-year ROC curves. **(G,H)** Data for 693 samples in CGGA. **(I,J)** Data for 325 samples in CGGA. ****p < 0.0001.

We further evaluated the predictive power of the prognostic model. The high and low groups are divided according to the median value of the risk score of the prediction model. In evaluating the predictive model from the TCGA data, there was a significant difference in the survival analysis, with an AUC value of 0.774 for the ROC curve in the third year (Figures 7A–F). Following that, we validate the dataset with an external source. The survival analysis was significantly different across the two CGGA data sets, with AUC values of 0.602 and 0.695 for the 3-year ROC curves, respectively (Figures 7G–J).

### Clinical information validates risk score as an independent prognostic factor

**FIGURE 8:**
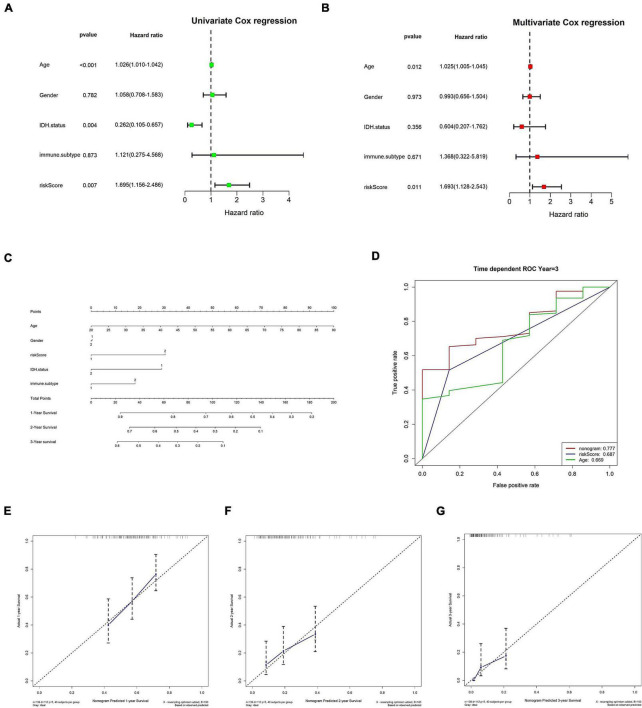
Clinical information was used to verify risk scores as an independent prognostic factor. **(A,B)** Independent prognostic factor analysis of risk scores. **(A)** Univariate Cox analysis. **(B)** Multivariate Cox analysis. **(C–G)** Nomograms. **(C)** Nomogram. **(D)** The nomogram’s 3-year risk score, stage, and overall ROC curve. **(E–G)** Calibration curves for 1, 2, and 3 years, respectively. In panel **(C)**, immune C1 in the immune. The subtype is 1, and immune C4 is 2; in gender, the male is 1 and female is 2; in IDH mutation, WT is 1 and mutant is 2; in risk score, low is 1 and high is 2.

Initially, both univariate and multivariate independent prognostic assessments of risk scores revealed substantial differences (Figures 8A,B). Then, using a nomogram and clinical features, we created a clinical practice guide. In the constructed nomogram, risk score and age had a more significant impact on prognosis, and the combined AUC value of risk score and clinical features in the ROC curve was 0.777 (Figures 8C–G).

## Discussion

One of the main characteristics of GBM is its great invasiveness of the surrounding nervous tissues, thus the investigation regarding certain proteins specifically expressed in the CNS, like the AQP4, should be more emphasized. Recent research indicate that GBM displays a pronounced genetic and biological heterogeneity between different tumors and even within the same tumor, which makes the tumor behavior highly variable and resistant to conventional therapy (Vitovcova et al., 2020). Thus, to find potential factors involved in tumor invasiveness and aggressiveness, it could be significantly important and informative to consider potential variation of molecular expression in human tumor samples. In the present study we evaluated the *AQP4*-related immunological pattern in various cancers, especially glioma. In particular, current bioinformatics analysis could be highly informative for further more in-depth research.

Previously, *AQP4* was well-descripted as brain-specific regulator and in recent years, there has emerged an intriguing and surprising link between *AQP4* and brain tumors (Ding et al., 2010; Yang et al., 2012; Zhao et al., 2012; Papadopoulos and Saadoun, 2015). Our previous research have also indicated the close involvement of *AQP4* in glioma cell invasion and migration, as well as drug resistance (Lan et al., 2017). In our previous study, we have also confirmed that *AQP4* was highly expressed in GBM tissues and that *AQP4* could impact glioma patients’ overall survival (Lan et al., 2020). However, the roles of *AQP4* in immunity have not been identified systematically. Current research provides a novel, vital *AQP4*-related immune status and prognostic model in glioma.

Tumor immunotherapy is a hot spot in the research field of various anti-tumor treatments (Chen and Mellman, 2017). The role of *AQP4* in immune regulation has not yet been reported. In recent years, many reports have identified the occurrence of the lymphatic system within the CNS and its essential role in immune regulation in brain tumor therapy. Therefore, this study further explored the role of *AQP4* in the immune regulation of glioma and the process of immunotherapy. Our study found that *AQP4* was significantly associated with the expression of multiple immune checkpoints and immune cells, and the *AQP4* expression level was negatively correlated with antigen presentation and processing capacity. Further, we combined literature reports and obtained enrichment scores through GSVA analysis and received the most relevant immunotherapy-related predictive pathways FGFR3 coexpressed genes, EGFR ligands, and pyrimidine metabolism. Furthermore, we found multiple immune cell effector genes significantly associated with *AQP4* expression levels. Although no significant correlation was found between the *AQP4* gene and IDH mutation and MGMT, *AQP4* had considerable expression differences in different immunophenotypes and molecular types. In addition, we further analyzed *AQP4*-related immunotherapy targets through the TTD database and found that in the GBM classification, the therapeutic targets significantly associated with *AQP4* expression were EGFR, ABAT, and PDGFRA. Finally, we screened the intersection of differential genes in the high and low *AQP4* gene expression group, the high and low immune score group, and the high and low matrix score group as candidate factors. The candidate genes were subjected to univariate cox analysis to obtain eight prognostic factors with significant differences. A clinical prognosis model constructed by three genes (RARRES1, SOCS3, TTYH1) was obtained through lasso dimensionality reduction. Finally, combined with clinical information and cox regression analysis, it was further confirmed that the model score could be used as an independent prognostic factor. The results of this study have a significant reference value for the prognosis research of *AQP4*-related glioma patients, and have indicated that *AQP4* could be considered as a potential new early biomarker of glioma progression (Frigeri et al., 2007; Du et al., 2020; Valente et al., 2022).

AQP4 protein is expressed as a particular morphological feature called orthogonal array of particles (OAPs). These structures are aggregates of the tetrameric unit (Valente et al., 2022). AQP4 protein is expressed as two major isoforms that differ in regard to methionine (M) starting codon. The shorter and more abundant form is called M23 and the longer and less abundant form is called M1 (Amiry-Moghaddam, 2019). The role of AQP4 isoforms in GBM biology has been addressed in earlier studies, which have shown that high-grade gliomas display higher expression of AQP4 than low-grade tumors (Warth et al., 2007). Furthermore, OAPs have been shown to be disintegrated or absent in GBM, and more recently an inverse correlation between OAP prevalence and malignancy was demonstrated (Fallier-Becker et al., 2016). The study by Simone et al. (2019) found that M1-AQP4 contributes to the invasiveness of glioma cells, while aggregation in OAPs by M23-AQP4 is deleterious and promotes apoptosis, interestingly indicating that the increased invasiveness was because of the increased activity of matrix metalloproteinase-9 (MMP9), which is associated with glioma cell proliferation and patient survival rate (Xue et al., 2017). Besides, AQP4 protein is expressed in different isoforms: two canonical M23 and M1 and two extended M23ex and M1ex, which influence expression, function and assembly in OAPs (Jin et al., 2011; Pisani et al., 2011; De Bellis et al., 2014). The extended isoforms are generated by the translational read through mechanism and are expressed in human CNS. Interestingly, Palazzo et al. (2019) found that research of AQP4ex-KO mice revealed that AQP4ex is indispensable to anchor AQP4 protein at the perivascular astrocytic end foot membrane domains. Indeed, large OAPs made of M1 and M23 canonical isoforms, still abundantly expressed in the AQP4ex mouse, are delocalized and confined at the astrocytic processes facing the brain neuropile. Thus AQP4ex may be supposed to be involved in the AQP4 alteration observed in the GBM (Valente et al., 2022). In the study of Valente et al. (2022), they evaluated the difference of expression and spatial distribution of AQP4 in grossly tumoral, peritumoral or non-tumoral brain regions. All these suggest that the AQP4ex isoform is critical in the triggering event of progressive downregulation and mislocalization of AQP4 in GBM, which may affect the integrity of the BBB, indicating that AQP4ex could be a potential early biomarker of GBM progression.

Currently, although kinds of immunotherapies have achieved remarkable success in cancer treatment, only limited number of patients could exhibit long-lasting anti-tumor response, where tumor immune infiltration status played a significant role (Chen et al., 2017). Identification of cancer patients with abundant infiltration of immune cells is of great importance to screen out potential candidates for immunotherapy. Our results of GBM and LGG cohorts highlighted immune-related GO and KEGG pathways in *AQP4* low- and high-expression groups, which along with results of the estimated immune infiltration level based on five algorithms could contribute to the distinction of “cold” and “hot” tumors. Our study then examined the immunological pattern of *AQP4* in gliomas, as well we the potential prognostic value of the *AQP4*-related signatures in the response of commonly used drugs and drug resistance of chemotherapy in different databases. Although *AQP4*-related prognostic model was consolidated with different public datasets, some more prospective and updated data are still necessary, and many genes for other traits that are of prognostic value may be excluded from the present study. We can conclude that *AQP4* could play a key role in the malignant proliferation and immunological regulation of human brain tumor. Besides, the barrier losing its normal microenvironment conformation may also be involved in the accumulation of edema in the peritumoral tissue (Valente et al., 2022). Thus *AQP4* could be considered as a potential new early biomarker of GBM progression (Frigeri et al., 2007; Du et al., 2020; Valente et al., 2022). The results presented in this study that could serve as a basis for increased research interest and warrant more detailed exploration. Recognition of *AQP4*-related therapy may open a new avenue for developing more specific targeted treatment for brain cancers. Our study provides robust evidence supporting *AQP4* as a new candidate for cancer treatment. Despite the prognostic value of the signature, this study still encountered several limitations which must be considered. Our report was retrospective and based on public databases, devoid of certain crucial clinicopathological information. Further biochemical experiments need to be conducted to confirm the findings. Furthermore, more work should be done to find factors determining AQP4 protein relocalization and AQP4 aggregation state in the near future, and this would accelerate definitive evaluation of the role of *AQP4* in the treatment of glioma and various other neurological diseases.

## Conclusion

The promoting role of *AQP4* in GBM cell invasion still need more research. Currently the significant roles of *AQP4* in combating drug resistance during glioma chemotherapy, as well as the potential AQP4 pharmacological blockers require additional research. We should pay close attention to the various unresolved questions regarding *AQP4* functions in brain tumors and various other CNS neurological diseases. It is noteworthy that most findings presented in this research are based on the bioinformatics techniques. Although the observations summarized in current study should be confirmed with more in-depth research, we believe that they could be critically informative for the design of more focused research in our future research, which will lead to definitive evaluation of the role of *AQP4* in the treatment of glioma and various other neurological diseases. For the first time, current research has examined the role of *AQP4* in the CNS immune system and find out how important it is in the glioma immunotherapy process. More importantly, we anticipated developing an *AQP4*-related prognostic model, which would serve as a critical theoretical research foundation for improving the effect of glioma immunotherapy. More effort should be directed toward clarifying the newly discovered functions and molecular mechanisms of *AQP4* in malignant gliomas. Furthermore, more in-depth explorations should be done to elucidate the roles of AQP4 protein relocalization in glioma in our future research.

## Data availability statement

The original contributions presented in this study are included in this article/Supplementary material, further inquiries can be directed to the corresponding authors.

## Author contributions

Y-LL and SZ contributed to conception, design, data processing, analysis, and interpretation. TN collected the data. SZ contributed to revision of the manuscript. All authors contributed to the manuscript and approved the submitted version.

## Footnotes

1
https://xenabrowser.net/

2
https://xena.ucsc.edu/

3
http://db.idrblab.net/ttd/

## Supplementary material

Supplementary Table 1 Twenty immune checkpoints used in current research.

Supplementary Table 2 The correlation analysis between the gene AQP4 and 20 immune checkpoints.

Supplementary Table 3 The correlation analysis between the gene AQP4 and 20 immune checkpoints (adjusted).

Supplementary Table 4 The original data regarding the connection between AQP4 gene and 22 immune invading cells.

Supplementary Table 5 The connection between AQP4 gene and 22 immune invading cells.

Supplementary Table 6 The connection between AQP4 gene and 22 immune invading cells (adjusted).

Supplementary Table 7 The original data regarding the association between the gene AQP4 and immune cells in various cancers.

Supplementary Table 8 The association between the gene AQP4 and immune cells in various cancers.

Supplementary Table 9 One hundred and twenty two genes collected from literature that potentially affects immune cells’ ability to present and process antigens.

Supplementary Table 10 The original data regarding the association between various immunological mechanisms and AQP4 gene expression.

Supplementary Table 11 Fourteen immunotherapy-related predictive pathways that could be significantly correlated with AQP4 expression.

Supplementary Table 12 The original data regarding the analysis of biological process downstream of AQP4 gene in glioma.

Supplementary Table 13 The original data regarding the analysis of cellular components downstream of AQP4 gene in glioma.

Supplementary Table 14 The original data regarding the analysis of molecular functions downstream of AQP4 gene in glioma.

Supplementary Table 15 The original data regarding the analysis of KEGG pathways downstream of AQP4 gene in glioma.

Supplementary Table 16 The original data regarding the association between tumor-associated immune cell effector genes and AQP4 gene levels.

Supplementary Table 17 The original data regarding the association between AQP4 expression levels and tumor clinical features.

Supplementary Table 18 The original data regarding the differential genes between the gene AQP4 high and low expression groups.

Supplementary Table 19 The original data regarding the divergent genes between the immunological score high and low groups.

Supplementary Table 20 The original data regarding the differential genes between the matrix score high and low groups.

Supplementary Table 21 AQP4 gene-related eight prognostic factors with great significance upon univariate cox analysis.

Supplementary Table 22 AQP4 gene-related three genes constructing the final scoring model upon multi-factor screening.
